# Supplementary material for: First insights into the Aurelia aurita transcriptome response upon manipulation of its microbiome
Source: Front Microbiol. 2023 Aug 10;14:1183627. doi: 10.3389/fmicb.2023.1183627 (PMC10448538; doi:10.3389/fmicb.2023.1183627)
Supplement: Supplementary file 5 [file Data_Sheet_1.PDF]

Supplemental Material

First insights into the *Aurelia aurita* transcriptome response upon manipulation of its microbiome

Nancy Weiland-Bräuer[2, #], Vasiliki Koutsouveli [1, #], Daniela Langfeldt[2, †], and Ruth A. Schmitz[2*]

Table S1: Transcriptomic data. (***A***) Number of raw and trimmed reads after filtering of native, sterile, and bacteria-challenged *Aurelia aurita* polyp pools. (***B***) Statistical data of the transcriptome assembly and the BLAST hits.

(***A***)

treatment	raw reads	filtered reads
native_1	15,410,175	13,701,069
native_2	15,248,818	13,492,120
*Klebsiella oxytoca*_1	12,989,916	11,351,244
*Klebsiella oxytoca*_2	11,099,887	9,576,528
*Vibrio anguillarum*_1	13,413,721	11,957,861
*Vibrio anguillarum*_2	15,393,642	13,528,712
*Pseudoalteromonas espejiana*_1	40,705,118	35,719,145
*Pseudoalteromonas espejiana*_2	16,786,302	14,696,662
AB-treated_1	6,873,558	5,320,279
AB-treated_2	11,639,511	10,234,034
AB-treated_3	7,708,609	6,168,876
Total	**167,269,257**	**145,746,530**

(***B***)

Counts of transcripts	
Total trinity 'genes'	**160,700**
Total trinity transcripts	**213,897**
GC content in %	**40**
**Statistics based on ALL transcripts**	
N10	3,574
N20	2,599
N30	2,003
N40	1,554
N50 **(contiguity score)**	**1,170**
Median contig length	390
Average contig length	710
Total assembled bases	**151,804,471**

N Transcripts with BLAST ID against Swiss-Prot	
**Total**	
**%**	
**Overall alignment rate in % (mapping/reproducibility score)**	**94.25**
**Annotation score in %**	**30.00**
**BUSCO Metazoa in % (n = 978)**	
**complete (completeness score)**	**96.42**
**single**	21.70
**duplicated**	74.70
**fragmented**	0.90
**missing**	2.70

Table S2: Annotation and Gene Ontology (GO) term identities of the whole transcriptome assembly.

TableS2.xlsx

Table S3: Differentially expressed genes (DE). Number of differentially expressed genes between the pairwise comparisons with their annotations and GO terms. (***Sample sheet A***) Number of genes that are up- or downregulated in AB-treated vs. native polyps. (***Sample sheet B - D***) Number of genes up- or downregulated in native polyps challenged for 30 min with 10^8 cells/mL of (***Sample sheet B***) *Klebsiella oxytoca,* (***Sample sheet C)*** *Vibrio anguillarum,* (***Sample sheet D***) *Pseudoalteromonas espejiana.* (***Sample sheet E - H***) Number of genes exclusively upregulated in native polyps challenged for 30 min with 10^8 cells/mL of (***Sample sheet E***) *Klebsiella oxytoca,* (***Sample sheet F***) *Vibrio anguillarum*, (***Sample sheet G***) *Pseudoalteromonas espejiana* compared to native polyps.

TableS3.xlsx

Table S4: Gene Ontology (GO) enrichment analysis of the differentially expressed genes. (***Sample sheet A***) enriched GO categories of upregulated genes in AB-treated polyps when compared to native polyps. (***Sample sheet B***) enriched GO categories of commonly upregulated genes in native polyps challenged for 30 min with 10^8 cells/mL of bacteria compared to native polyps. (***Sample sheet C-E***) enriched GO categories of exclusively upregulated genes in native polyps challenged for 30 min with 10^8 cells/mL of (***Sample sheet C***) *Klebsiella oxytoca,* (***Sample sheet D***) *Vibrio anguillarum,* (***Sample sheet E***) *Pseudoalteromonas espejiana* compared to native polyps.

TableS4.xlsx

Table S5: Assembly information of function-based identified QQ-ORFs of *A. aurita*.

TableS5.xlsx
